# Soil Moisture Is the Key Factor Facilitating Giant Ragweed Invasions in Grasslands of the Yili Vally, China

**DOI:** 10.3390/biology14030249

**Published:** 2025-02-28

**Authors:** Xinyi Chen, Zhanli Song, Baoxiong Chen, Wanli Yu, Hegan Dong

**Affiliations:** 1 Production and Construction Corps Key Laboratory of Oasis Town and Mountain-Basin System Ecology, Shihezi University, North 4 Rd., Shihezi 832003, China; chenxinyi@stu.shzu.edu.cn; 2 College of Life Sciences, Shihezi University, North 4 Rd., Shihezi 832003, China; 3 College of Water and Architectural Engineering, Xinjiang Shihezi Vocational and Technical College, No. 38 Community, Shihezi 832003, China; 4 Agricultural Ecology and Resource Protection Station of the Ministry of Agriculture and Rural Affairs, Beijing 100125, China; 5 Xinjiang Uygur Autonomous Region Agricultural Ecology and Resources Protection Station, Urumqi 830000, China; 6 Western Agricultural Research Center, Chinese Academy of Agricultural Sciences, Changji 831100, China

**Keywords:** giant ragweed, temperate grassland, population distribution, interspecific competition, soil water, prevent and control

## Abstract

Giant ragweed (GR; *Ambrosia trifida* L.), an invasive alien species, causes significant harm to grassland ecosystems and farmlands in some areas but is challenging to control. GR has invaded the hilly grasslands of Yili Valley, China, since 2013, and preliminary observations have shown that GR populations on the lower slopes of hills are more successful than those on the middle or upper slopes. To clarify the factors determining GR’s invasion success, we compared GR population distributions among slope positions and the relationship between non-biotic factors and the invasion of GR. Of the soil physicochemical properties, only soil moisture differed significantly among slope positions, with the wettest soils found on the lower slopes. GR biomass increased with the soil water content, irrespective of native plant diversity. In our experiment, when the annual average soil volume moisture content exceeded 20.3% and 25.3%, GR could reduce the biomass of native herbs by more than 50% and 80%. Therefore, water is the determining factor of a successful GR invasion in the grasslands of the Yili Valley. On a global scale, it was discovered for the first time that GR can invade temperate grasslands, but also has risks of invading other grasslands that share similar conditions. So, GR invasions of temperate grasslands must be closely monitored, particularly in low-lying areas or those with increasing precipitation.

## Simple Summary

Giant ragweed is a worldwide invasive species; it started to threaten the Yili Valley grassland in 2013. To make a better control plan, it is vitally important to find the decisive factors of GR invasion. After several years of systematic o bservation, we found that GR exhibits the most vigorous growth at the foot of the mountain. We investigated three subregions of mountain steppe and measured and analyzed the physical and chemical properties of underground soil in each region. It was confirmed that GR had higher plant height, cover, and biomass at the lower slope. The soil moisture content was highest in the bottom slope. In addition, the analysis of our experimental results showed that the higher the soil moisture, the higher the GR biomass, and there was a strong correlation between these two indexes. The water content is the key to the success of GR invasion.

## 1. Introduction

Giant ragweed (GR; *Ambrosia trifida* L., family *Asteracea*), which is native to North America, is an invasive annual plant that is found in more than 40 countries worldwide [1,2,3,4]. It is characterized by early germination, fast growth, unusual height, large quantities of seeds, and high population density [5,6,7]; these characteristics contribute to the high invasiveness of this species [8,9]. GR is widely distributed in a variety of habitats, including roadsides, farmlands, wastelands, forests, and residential areas [6,10,11,12]. In farmland, GR populations reduce crop and forage yields, and GR pollen poses a threat to human health as a major cause of hay fever [13,14,15]. In Europe, ragweed-associated health problems are estimated to lead to annual economic losses of more than EUR 6.2 billion [16]. For convenience, abbreviations are used throughout this paper.

Grasslands are the predominant terrestrial ecosystem globally, accounting for 24.58% of the total area of land on Earth [17]. The diversity–invisibility hypothesis suggests that the more diverse a plant community is, the more resistant it is to invasive plants [18,19]. As grasslands are typically rich in plant diversity, the “diversity resistance hypothesis [20]” suggests that external species should find the grassland ecosystem difficult to invade [21,22]. However, we have previously observed that GR first invaded the grasslands of Yili Valley, Xinjiang Autonomous Region, China, in 2013; by 2019, GR was distributed across more than 11, 900 hectares of the Yili Valley, a 238-fold increase in the distribution area in seven years [23]. In the Yili Valley, GR poses a serious threat to grassland biodiversity, human and animal health, and tourism [7], but GR control in grasslands is more complicated than in farmland because the chemical herbicides used to control GR also harm other herbs and livestock [7]. Therefore, the control of GR in grasslands is challenging.

The Yili Valley grasslands are a temperate, semi-arid region with an average annual temperature of 10.4 °C and an average annual precipitation of 400–600 mm [24]. Because it is clear that GR easily invades and thrives over large areas of temperate grasslands in the Yili Valley, ecologically similar grasslands elsewhere in the world may also be at risk of GR invasion.

There are many factors that determine how likely plants are to become invasive in a given habitat, primarily including the invasiveness of the plant, the susceptibility of the habitat, and human interference [25,26,27]. The invasiveness of a potential invader refers to the invasion fitness of potentially invasive plants with certain traits in a saturated community; low invasion fitness indicates high similarity between potential invasive plants and local species in an unsaturated community, and this corresponds to low invasiveness [28]. Many characteristics of invasive species, either intrinsic or extrinsic to them, have been implicated in their success in novel environments [29,30]. Traits intrinsic to invasive plants generally represent the life history/morpho-functional traits of the invading species, which include high growth rates, reproductive fertility, the ability to disperse, plasticity, and allelopathy [31,32,33]. On the other hand, traits extrinsic to invaders involve environmental characteristics of the invaded habitat, such as topography, biological diversity, the characteristics of the interacting native vegetation, resource availability, disturbance levels, and lack of natural enemies [34,35]. Disturbance is considered an important extrinsic factor in a habitat’s susceptibility to invasion [36]. The susceptibility of a habitat is affected both by the abiotic environment [37] and the biotic environment [38]. The abiotic environment includes water, temperature, and soil physicochemical properties [39], while the biotic environment includes the biodiversity and interspecific competitiveness of indigenous species [40]. For grasslands with relatively low levels of human disturbance and resource fluctuation, plant invasiveness depends on plant competition mechanisms [41,42] and propagule pressure [43,44]. Although GR has intense intraspecific competition, when its population density reaches the highest value without self-suppression, it still has the potential to occupy a large area [45]. That is to say that when GR invades a habitat, and its population density reaches a certain threshold, it will expand across that habitat, occupy the ecological niches of local species, and gradually form a single-dominant species community. As the invasion time increases, GR can change its morphological, physiological, biochemical, and other phenotypic plasticity characteristics to adapt to local habitat conditions [46].

**Figure 1:**
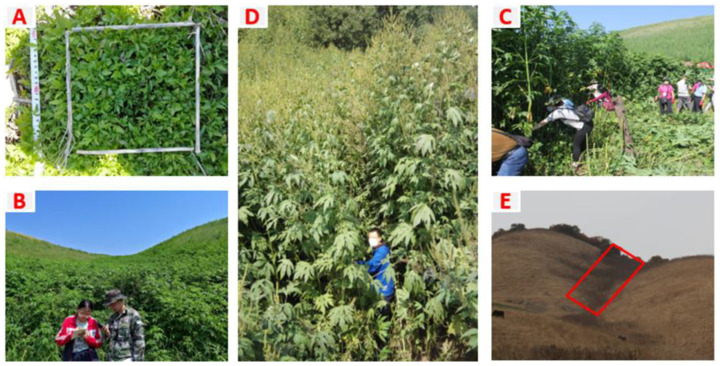
Population growth of giant ragweed (*Ambrosia trifida* L.) at the seedling period, growing period, and mature period. (**A**–**E**) are taken in the same place as the grassland of Yili Vally, respectively, showing the population growth of giant ragweed during these periods. (**A**–**D**) Population growth can be seen at the bottom of the slope, with the green part showing the giant ragweed population. The dark gray in the red box of the bottom slope in (**E**) is the giant ragweed population, and there is no obvious giant ragweed population in the middle and top slope. The above photos were taken in 2019.

In addition, GR abundance in grasslands varies with local topography; in hilly terrain, GR is widely distributed at the bottom slope, with population density gradually decreasing with elevation from the bottom slope (BS) to the middle slope (MS) and top slope (TS) [7] (Figure 1). Therefore, it is necessary to thoroughly characterize the invasion mechanisms of this plant and determine the factors driving the success of GR invasion in grasslands, which plays an important role in its precise prevention and control. Hilly grasslands are a useful study area; in this study, our main objective was to identify the important factors driving the success of GR within hilly grasslands. To achieve this objective, we had three main aims as follows: (1) to measure the distribution of GR across various slope positions; (2) to analyze the competition between GR and native herbs at different slopes; and (3) to investigate how soil properties, temperature, and moisture impact GR growth and reproduction in the Yili Valley grassland. We will summarize the factors that determine the success of GR invasion in grassland.

## 2. Materials and Methods

### 2.1. Research Area and Sample Plot Setting

The study area was located in the Yili Valley grasslands (42°14′16″–44°53′30″ N, 80°09′42″–84°56′50″ E) in the Xinjiang Autonomous Region, China. The grassland is mainly used for grazing, and hills are the main topographical feature [7].

**Figure 2:**
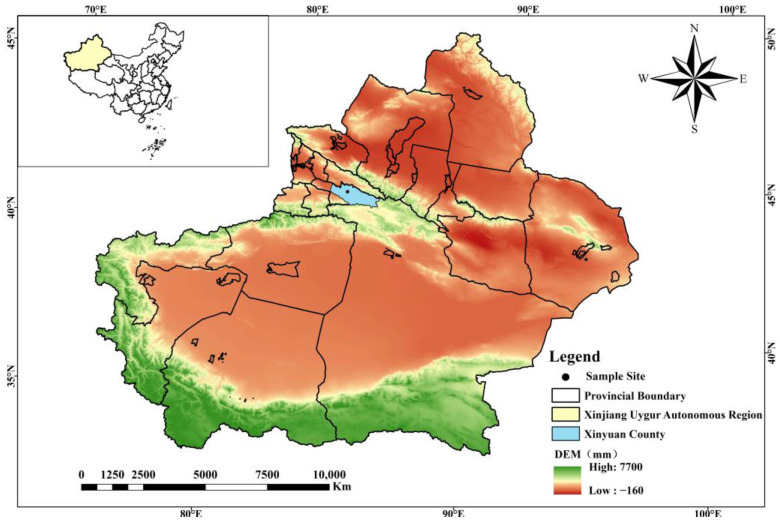
Location of the Yili Valley and sample plot within the Xinjiang Autonomous Region.

Four years after its invasion in the Yili Valley (i.e., 2017), the GR population stabilized in local areas [7]. In March 2019, nine sample plots (each 5 m wide × 15–30 m long) were established in the Yili Valley grasslands. In 6 plots (plots 1–6), GR had been growing for five years. The remaining three plots (plots 7–9) were free from GR. Plots were at least 1 km apart in all directions, and each plot included areas of the BS, MS, and TS. All slopes were 20–30° (Figure 2).

### 2.2. Taxonomy of Native Herbs in Plots Without GR Invasion

In May, July, and September 2019, three quadrats of 3 m × 3 m were set up on the BS, MS, and TS of plot 7, plot 8, and plot 9, respectively, and the species of native herbs were counted. If a species appeared at a certain slope position at any time, it was considered that the species was at that slope position. We counted the taxonomy and life cycles of native herbs in all slope positions.

### 2.3. Growth and Reproduction of GR and Native Herbs at Different Slope Positions

#### 2.3.1. Population Density, Coverage, and Plant Height

The numbers of native herbs and GR plants in the BS, MS, and TS areas of each plot were counted three times during the GR growing season in 2019: 20–22 April, during the GR seedling period; 10–12 June, during the GR growing period; and 10–12 September, at GR maturity. The heights of 30 randomly selected GRs and native herbs were measured in each slope area in each plot; for plots with fewer than 30 plants of either type, all plants were measured.

#### 2.3.2. Biomass

During the third counting event (10–12 September 2019), 1 m × 1 m quadrats were established in each of the BS, MS, and TS areas within each of the nine sampling plots. GR and other herbaceous plants in each quadrant were collected, air-dried, and weighed separately using an electronic balance (BDS, Shanghai, China) to calculate the biomass of GR and native herbs per unit area.

#### 2.3.3. Seed Quantity

During the third counting event (10–12 September 2019), 1 m × 1 m quadrats were established in each of the BS, MS, and TS areas within each of the six GR-populated sampling plots (plots 1–6). These plots were distinct from those used for biomass sampling. Six GR plants per quadrat were randomly selected, and all seeds on each plant were counted and removed. If some seeds had fallen, we estimated the seed quantity based on the locations of the seeds [6].

### 2.4. Soil Physicochemical Properties, Temperature, and Moisture at Different Slope Positions

#### 2.4.1. Soil Physicochemical Properties

In April 2019, soil samples (0–10 cm depth) were taken at the TS, MS, and BS of sample plots 1–6 (18 samples in total). Total nitrogen, total phosphorus, and total potassium in these samples were determined using the micro-Kjeldahl, sodium hydroxide melting-molybdenum anti-colorimetry, and flame photometry methods [47], respectively. Available nitrogen, phosphorous, and potassium were measured using the alkaline hydrolysis diffusion method, Mo-Sb colorimetry, and the ammonium acetate method, respectively [48]. Soil pH was measured using a Mettler-Toledo pH meter (UB−10, Columbus, OH, USA), and soil conductivity was measured using a conductivity meter (Hach, Loveland, CO, USA). Soil organic matter content was calculated using the K_2_CrO_7_-H_2_SO_4_ external heating method.

#### 2.4.2. Soil Temperature and Moisture

A soil temperature and moisture meter (Watch Dog 1200, Spectrum Technologies, Inc., Aurora, IL, USA) was placed in the uppermost 10 cm soil layer in each TS, MS, and BS area of sample plots 1–6 on 20 March 2019; the meters were removed on 2 April 2020. Meters recorded data every hour. Temperature and moisture data were divided into four groups: the seedling period (1 April–31 May 2019), the growing period (1 June–31 July 2019), the mature period (1 August–30 September 2019), and the winter season (1 October 2019–31 March 2020). We calculated the average soil temperature and moisture within each period and the relative frequency of soil temperature and moisture throughout the growing season (1 April–30 September 2019). We also calculated average soil moisture throughout the year (1 April 2019–31 March 2020).

### 2.5. Effects of Water on the Growth and Reproduction of GR

A plant growth experiment was performed in the experimental garden from October 2017 to October 2018 in Yining City (43°50′–44°09′ N, 80°04′–81°29′ E), located in the Yili Valley. This locale has an average annual temperature of 10.5 °C and an average annual precipitation of 280 mm. Three irrigation treatment gradients were established in the experimental garden: no irrigation with 280 mm of annual precipitation; 2800 m^3^/hm^2^ of irrigation during the growth period (equivalent to 560 mm of annual precipitation supplemented by 400 m^3^/hm^2^ of irrigation every month from April to October 2018), and 5600 m^3^/hm^2^ of irrigation during the growth period (equivalent to 840 mm of annual precipitation supplemented by 800 m^3^/hm^2^ of irrigation every month from April to October 2018). Each water treatment was tested with three plots, and nine plots were randomly arranged with 3 m × 3 m plots for each irrigation sample area. Plastic film was buried vertically to a depth of 40 cm in the soil around each irrigation plot to separate the water received in each plot. Each plot was uniformly sprinkled with 900 seeds of GR.

The density and plant height of GR were observed during the seedling period (15 May), growing period (15 July), and mature period (20 September) in 2018, and seed yield per m^2^ was observed in the mature period (20 September) in 2018.

### 2.6. Statistical Analysis

All data were analyzed using SPSS 20 (IBM, Armonk, NY, USA) and visualized using OriginPro 8.5 (OriginLab, Northampton, MA, USA). One-way analysis of variance (ANOVA) and multiple least significant difference (LSD) comparisons were used to explore the differences among slope positions with respect to GR biomass, seed quantity, population density, coverage, and plant height; native herb population density, coverage, and plant height; and soil physicochemical properties, temperature, and moisture.

We also compared native herb population density, coverage, height, and biomass in sites with GR to sites without GR. We also compared the population density, height, and seed yield per m^2^ of GR under different moisture conditions.

PCA and heat maps were used to explore how well soil-available nitrogen, available phosphorus, available potassium, organic matter, conductivity, pH, native-herb biomass, average temperature, and moisture predicted GR biomass and seed quantity throughout the growth period. Linear regression analysis was used to determine the relationship between annual average soil volume moisture and the percent reduction in native herb biomass per unit area in the GR-invaded sites.

## 3. Results

### 3.1. Interspecific Coexistence Between GR and Native Herbs in Different Slope Positions

Yili Valley grasslands harbor substantial plant diversity. In addition to GR, we identified 26 species in 26 genera and 12 families across all sites. We identified all 26 species at BS sites (10 annual herbs, 2 biennial herbs, and 14 perennial herbs), 23 species at MS sites (9 annual herbs, 2 biennial herbs, and 12 perennial herbs), and 16 species at TS sites (5 annual herbs, 1 biennial herb, and 10 perennial herbs). Thus, native-herb species richness was greatest for the BS, followed by the MS and TS (Table 1).

**Figure 3:**
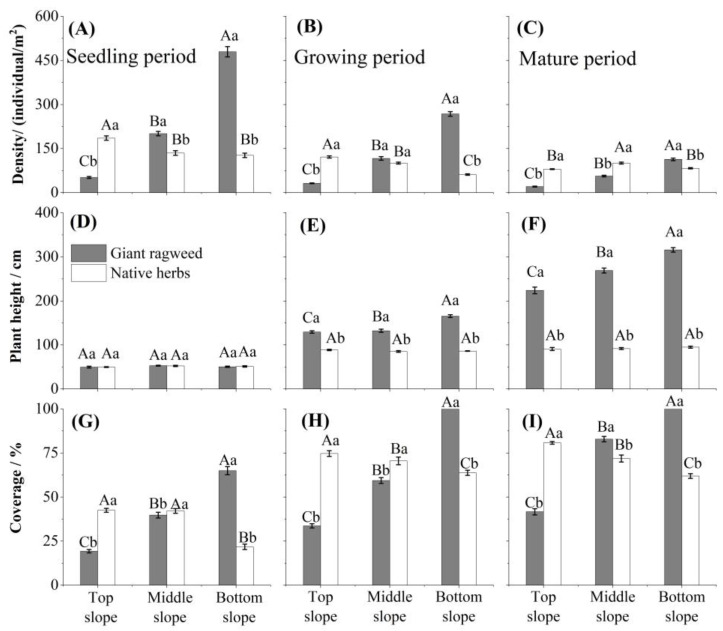
The population density, plant height, and population coverage of giant ragweed (*Ambrosia trifida* L.) and native herbs during the seedling period, the growing period, and at maturity at different positions on slopes in Yili Valley, China. Different capital letters indicate significant differences among slope positions (*p* < 0.05, least significant difference test). Different lowercase letters indicate significant differences between giant ragweed and native herbs (*p* < 0.05, least significant difference test). (**A**,**D**,**G**) A comparative analysis of the density, plant height, and coverage of native species at different slope positions as well as giant ragweed during the seedling stage. (**B**,**E**,**H**) A comparative analysis of the density, plant height, and coverage of native species at different slope positions as well as giant ragweed during the seedling stage. (**C**,**F**,**I**) A comparative analysis of the density, plant height, and coverage of native species at different slope positions as well as giant ragweed during the seedling stage.

Native herb population density and coverage were generally inversely proportional to GR population density and coverage; in the TS, native herbs were significantly denser and had significantly greater coverage than GR throughout the growing season (*p* < 0.01) (Figure 3A–C,G–I). However, native herbs remained significantly shorter than GRs at all sites during the growing period and at maturity (Figure 3E,F).

### 3.2. Effects of Soil Physicochemical Properties, Temperature, and Moisture on GR Growth and Reproduction

**Figure 4:**
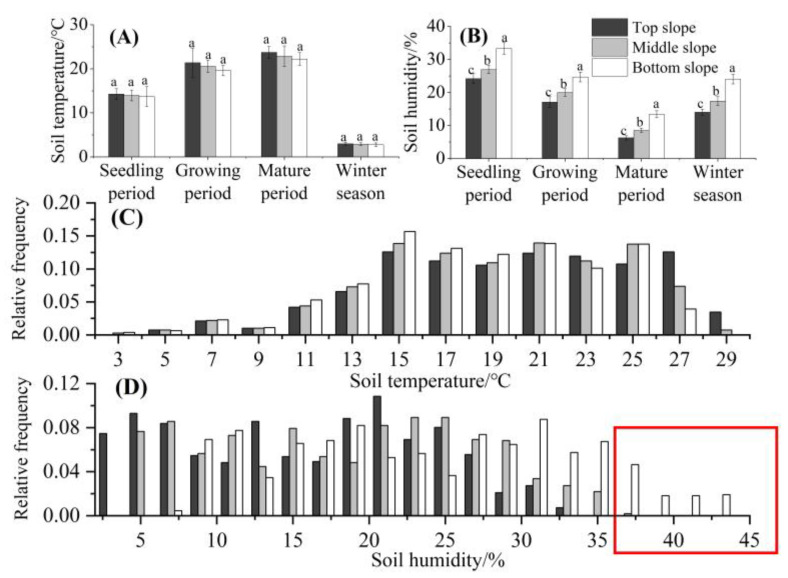
Soil temperature and moisture during the seedling period, growing period, mature period, and winter season at different positions on slopes in the Yili Valley, China. Different letters indicate significant differences among the slope positions (*p* < 0.05, least significant difference test). (**A**) Soil temperature at different slope positions in different growth stages of GR. (**B**) Soil humidity at different slope positions in different growth stages of GR. (**C**) The relative frequency of GR in relation to soil temperature variations at different slope positions. (**D**) The relative frequency of GR in relation to soil humidity variations at different slope positions. The role of red frame is to show that GR occurs more frequently when the water content is greater than 35%.

There were no significant differences in soil physicochemical properties (soil total nitrogen, total phosphorus, total potassium, available nitrogen, available phosphorus, available potassium, organic matter, pH, and conductivity) among the three slope positions (Table 2). The soil temperature also did not differ significantly among slope positions at any point during the year (Figure 4A). However, soil moisture differed significantly among slope positions at each measured point during the year, with the TS being significantly less moist than the MS and the MS being significantly less moist than the BS (*p* < 0.01). Soil moisture levels at the TS, MS, and BS were 24.1%, 26.9%, and 33.3%, respectively, in the seedling period; 17.1%, 19.9%, and 24.7%, respectively, in the growing period; 6.3%, 8.5%, and 13.4%, respectively, during the mature period; and 14.0%, 17.4%, and 24.1%, respectively, in the winter season (Figure 4B). The relative frequency of soil temperature during the GR growing period between different slope positions had little difference (Figure 4C). The relative frequency of soil moisture at the BS in the GR growing period had obvious differences compared to the MS and TS, and the soil moisture had a greater distribution frequency of 38–44% (better moisture condition) at the BS (Figure 4D).

**Figure 5:**
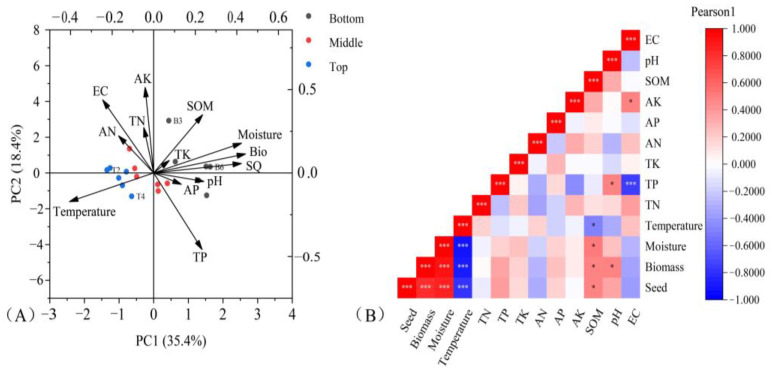
(**A**) PCA of biomass and habitat soil factors of *Ambrosia trifida* L. (**B**) A heat map. AN, available nitrogen (g/kg); AK, available potassium (g/kg); AP, available phosphorus (g/kg); TN, total nitrogen(g/kg); TK, total potassium (g/kg); TP, total phosphorus (g/kg); pH, acidity; EC, electrical conductivity (us/cm); SOM, soil organic matter; SQ, seed quantity per m^2^; Bio, biomass per m^2^; T, the top of the mountain; M, the middle of the mountain; B, the bottom of the mountain. “*”, *p* < 0.05; “***”, *p* < 0.001.

We performed principal component analysis (PCA) and heatmap analysis on the soil physical and chemical factors, biomass, and seed yield data of GR. The results revealed the fact that soil moisture and organic matter content (SOM) exhibited a positive correlation with PC 1 and PC 2 (Figure 5A). Furthermore, the soil moisture and organic matter content had a positive correlation with the biomass and seed quantity of GR. The correlation between soil moisture and biomass (r = 0.92) was stronger than that between soil moisture and seed amount (r = 0.85) (Table S1). Additionally, temperature showed a significant negative correlation with these two indexes (r _Biomass_ = −0.89, r _Seed_ = −0.78) (Figure 5B).

### 3.3. The Water Demand for Plant Growth and Reproduction in GR

GR grew better under 560 mm and 840 mm of simulated annual precipitation than under 280 mm of annual precipitation during the growing period. When comparing plants experiencing 840 mm of simulated rainfall and 280 mm of annual precipitation, the density, plant height, and seed yield of GR decreased by 88.5%, 74.5%, and 99.9%, respectively, in the mature period (Table 3).

### 3.4. Negative Effects of GR on Native Herbs in Grassland Areas with Different Moisture Levels

**Figure 6:**
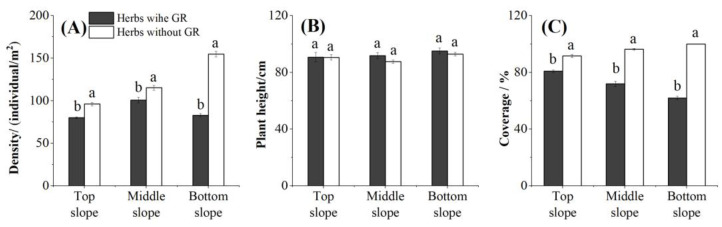
Native herb density, coverage, height, and biomass per unit area at different positions on the slopes in Yili Valley, China, for sites with and without giant ragweed (*Ambrosia trifida* L.). Different letters indicate significant differences between native herb populations growing with and without giant ragweed (*Ambrosia trifida* L.; *p* < 0.05, least significant difference test). (**A**) Comparison of native herb density between plots with and without GR invasion. (**B**) Comparison of native herb height between plots with and without GR invasion. (**C**) Comparison of native herb coverage between plots with and without GR invasion.

**Figure 7:**
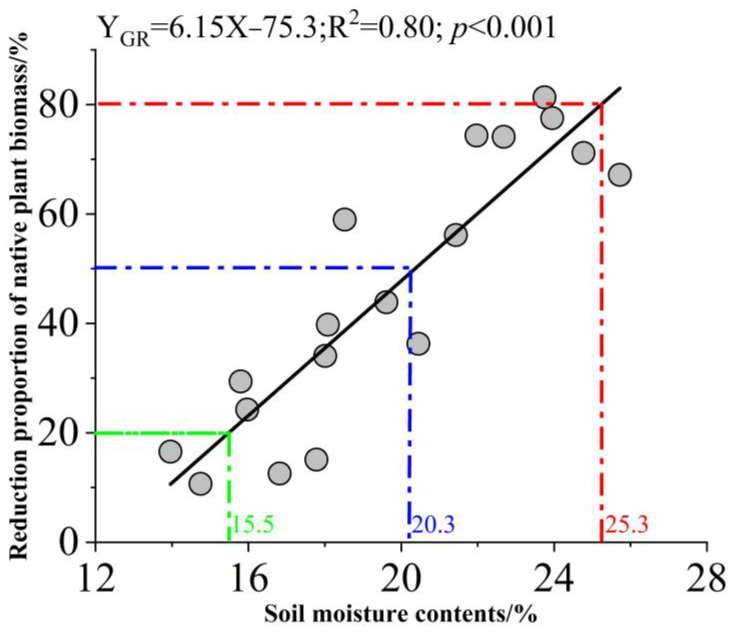
The linear relationship between annual average soil moisture content and reduction in native plant biomass yield per unit area in areas of Yili Valley, China, colonized by giant ragweed (*Ambrosia trifida* L.). The green dotted line represents the annual average soil moisture content corresponding to a 20% reduction in native herb biomass (similar to soil moisture levels at the top slope of plots 1–6), the blue dotted line represents the annual average soil moisture content corresponding to a 50% reduction in native herb biomass (similar to the soil moisture levels at the middle slope of plots 1–6), and the red dotted line represents the annual average soil moisture content corresponding to an 80% reduction in native herb biomass (similar to the soil moisture level at the bottom slope of plots 1–6).

The native flora of plots colonized by GR for ≥5 years differed noticeably from that of as-yet uninvaded plots. Across all slope positions, native herb population density and coverage were significantly lower in the invaded plots compared to the uninvaded plots; native plant height did not differ between the two sets of plots (Figure 6B). The TS, MS, and BS population densities of the invaded plots were 16.8%, 12.6%, and 46.3% lower than the uninvaded plots, respectively, while population coverage was 11.8%, 25.3%, and 38.1% lower (Figure 6A,C). Interestingly, as soil moisture increased, the difference in native plant biomass between the invaded and uninvaded sites became more obvious. At a soil moisture content of 15.5%, native herb biomass was 20% lower in the invaded sites (primarily in the TS of plots 1–6); at a soil moisture content of 20.3%, native herb biomass was 50% lower in the invaded sites (primarily in the MS of plots 1–6); and at a soil moisture content of 25.3%, native herb biomass was 80% lower in the invaded sites (primarily in the BS of plots 1–6; Figure 7).

## 4. Discussion

### 4.1. Factors Determining the Success of GR Invasion in Temperate Grasslands

By 2017, five years after GR invaded the hilly grasslands of the Yili Valley, the GR population had reached a relatively stable state in the local area [7]. Here, we observed that from the BS to the TS, GR population density, coverage, plant height, and biomass decreased significantly, indicating that GR populations tended to be dense at the BS and sparse at the TS (Figure 3). GR is typically a vigorous competitor due to its early germination, rapid growth, and large seed yield, which, together, allow the plant to outgrow competitors, develop superior biomass, and attain high levels of coverage using Soil Agricultural Chemical Analysis Methods [7,11,13,49]. Generally, invasive plants more easily invade areas with low species diversity but harder to invade species-rich habitats, because stronger species interactions limit the establishment and spread of exotic species [50]. However, GR population density and coverage were significantly greater at the BS compared to the native herbs, while the reverse was true at the TS (Figure 3). Interestingly, native-herb species richness was highest at the BS and lowest at the TS (Table 1).

GR requires sufficient water to support its interspecific competitive advantage [45,51,52]. In the Yili Valley, the only significant difference in the measured abiotic properties among the BS, MS, and TS was soil moisture (Table 2 and Figure 4). That is, the BS sites had appropriate levels of soil moisture for GR growth, which supported increases in GR population density, coverage, plant height, and seed yield and allowed GR to outcompete native herbs at these sites. However, the soil moisture levels at the MS and TS sites were insufficient for optimal GR growth, weakening the interspecific competitiveness of GR and preventing this invasion from outcompeting the native herbs. Consistent with this, GR populations in farmlands, roadsides, and residential areas are frequently located in low-lying and waterlogged areas. Indeed, GR populations worldwide generally thrive in habitats with relatively high moisture levels, such as riverbanks and ports [53]. Soil moisture was significantly correlated with GR biomass and seed quantity per unit area in this study (Figure 5A). The field experiment showed that the growth and reproduction of GR were strongly water-dependent. When the water was insufficient, the population density and seed yield decreased significantly (Table 3). Therefore, soil water content may also be important for GR invasions in other temperate grassland areas worldwide. Similar findings were recovered in previous studies [6,54]. Notably, despite the relatively high diversity of grassland plants, GR invasions are likely to be successful given sufficient soil moisture.

Soil provides the necessary material basis for the survival and development of vegetation [55]; soil physical and chemical properties (soil total nitrogen, total phosphorus, total potassium, available nitrogen, available phosphorus, available potassium, organic matter, pH, and conductivity) are important components of soil health, which influence water and nutrient movements, aeration, soil temperature, nutrient cycling, and root growth to affect the normal plants growth [56]. Each of them has the potential to be a limiting factor for plant growth in habitats. Though soil organic matter and temperature show a positive/negative correlation with biomass and seed amount in PCA, in our study, there was no significant difference in organic matter content and temperature at different slope sites, and GR formed a single-dominant species community at the bottom of the slope, indicating that soil organic matter and temperature were not in the “inhospitable” range of ragweed in the hilly steppe of Yili Valley.

Grassland plant community productivity is a key issue in grassland protection [57]. GR strongly inhibits the colonization and growth of other annual plants. Our results suggest that GR also deleteriously affects biennial and perennial herb populations at the BS: at annual average soil moistures of 15.5–25.3%, the biomass of native herbs (annuals, biennials, and perennials) decreased 20–80% 5 years after GR invasion compared to plots free of GR. Hartnett et al. showed that GR negatively affects most woody plants, including wild apricots, as well [58]. Thus, GR growth may lead to significant declines in the community productivity of both native herbs and woody plants, and the most reliable predictor of the success of GR invasion, as well as the degree of GR-associated damage, is the soil moisture level.

### 4.2. Prevention and Control Suggestions

Except for the Yili Valley, no other grasslands are known to have been invaded by GR on a large scale. However, most grasslands worldwide are temperate, with similar climatic conditions and levels of rainfall to the Yili Valley. Climate change is also expected to increase extreme weather events in temperate zones, for example, increasing the frequency of heat waves, which will affect plant growth and reproduction during the whole life cycle [59,60,61]. Thus, these temperate grasslands may be under threat of GR invasion, particularly if rainfall increases due to climate change. Proposed methods for GR control in grasslands must consider both present levels of soil moisture and the possible effects of increased rainfall in the future.

Because each GR plant can produce an average of 450 seeds [7], and in grassland soil, the seed bank can reach an average of 10,000 seeds/m^2^ [6], each of which may remain viable for more than 9 years [62], it is difficult to completely control GR once it invades. Our results suggest that GR easily invades temperate grasslands with sufficient rainfall or, if rainfall is insufficient, grassland catchment areas, such as the BS and under trees [63]. Therefore, it is critical to monitor and control GR growth in these areas. Moreover, our results suggest that, unlike some other invasive plants [64,65], plant substitutions cannot be used as the main method of GR control in temperate grasslands. As long as there is sufficient soil moisture or water supply, GR is likely to become the dominant plant species soon after the initial invasion (~4 years), even if plant biodiversity is rich and interspecific competition is strong.

Once grassland biodiversity is destroyed, it is very difficult to restore [7]. In the Yili Valley grasslands, the native herbs are mainly perennial species of the *Poaceae* [66]. However, once these plants are outcompeted by the invading GR, they are unlikely to return; even after a complete eradication of GR, the empty ecological niche is likely to be reoccupied by non-foraging species such as GR or *Cannabis sativa* L. Therefore, GR prevention and control in grasslands should focus on monitoring to prevent initial invasion and the rapid extermination of any identified invaders.

Even if GR is completely removed, the vacant niche may be occupied by other species

## 5. Conclusions

Soil water content is a decisive factor in GR invasion into temperate grassland. GR biomass per unit area increased with soil water content, irrespective of native plant diversity. When the annual average soil volume moisture content exceeds 20.3% and 25.3%, the GR can reduce the biomass of native herbs by more than 50% and 80%. Therefore, GR invasions of temperate grasslands must be closely monitored, particularly in low-lying areas or those with increasing precipitation.

## Figures and Tables

**Table 1:** Taxonomy and life cycles of native herbs found at different positions on slopes in Yili Valley, China: top slope, middle slope, and bottom slope.

Family	Genus	Species	Life Cycle	Top Slope	Middle Slope	Bottom Slope
Compositae	*Erigeron*	*Erigeron canadensis* L.	Annual	×	√	√
	*Xanthium*	*Xanthium sibiricum* L.	Annual	×	×	√
	*Taraxacum*	*Taraxacum mongolicum* Hand.-Mazz.	Perennial	√	√	√
	*Cirsium*	*Cirsium arvense var. integrifolium* Wimm. and Grab	Perennial	√	√	√
	*Achillea*	*Achillea millefolium* L.	Perennial	×	×	√
	*Cichorium*	*Cichorium intybus* L.	Perennial	√	√	√
	*Artemisia*	*Artemisia vulgaris* L.	Perennial	×	×	√
	*Sonchus*	*Sonchus oleraceus* L.	Biennial	×	√	√
Moraceae	*Cannabis*	*Cannabis sativa* L.	Annual	√	√	√
Leguminosae	*Trifolium*	*Trifolium* L.	Perennial	√	√	√
	*Medicago*	*Medicago sativa* L.	Perennial	√	√	√
	*Sophora*	*Sophora alopecuroides* L.	Annual	√	√	√
Umbelliferae	*Daucus*	*Daucus carota* L.	Biennial	√	√	√
Amaranthaceae	*Amaranthus*	*Amaranthus retroflexus* L.	Annual	×	√	√
Chenopodiaceae	*Chenopodium*	*Chenopodium album* L.	Annual	×	√	√
Poaceae	*Bromus*	*Bromus japonicus* Thunb.	Annual	√	√	√
	*Echinochloa*	*Echinochloa crusgalli* (L.) P. Beauv.	Annual	√	√	√
	*Setaria*	*Setaria viridis* (L.) P. Beauv.	Annual	√	√	√
	*Elytrigia*	*Elytrigia repens* (L.) Nevski	Perennial	√	√	√
	*Poa*	*Poa annua* L.	Annual	√	√	√
	*Agrostis*	*Agrostis clavata* Trin.	Perennial	√	√	√
Rosaceae	*Potentilla*	*Potentilla chinensis* Ser.	Perennial	×	√	√
Labiatae	*Phlomis*	*Phlomis umbrosa* (Turcz.) Kamelin and Makhm.	Perennial	√	√	√
Urticaceae	*Urtica*	*Urtica fissa* E. Pritz.	Perennial	√	√	√
Boraginaceae	*Trigonotis*	*Trigonotis peduncularis* (Trevis.) Benth. *Moore*	Annual	×	√	√
Cyperaceae	*Carex*	*Carex obtusata* Lilj.	Perennial	×	√	√

√: the species is distributed on the slope; ×: the species is not distributed on the slope.

**Table 2:** Soil physical and chemical properties at different positions on slopes in Yili Valley, China.

Index	Top Slope	Middle Slope	Bottom Slope
Total nitrogen/(%)	12.87 a	12.77 a	12.87 a
Total phosphorus/(g·kg^−1^)	0.07470 a	0.07450 a	0.07681 a
Total potassium/(g·kg^−1^)	21.52 a	21.43 a	21.90 a
Available nitrogen/(mg·kg^−1^)	2.080 a	2.060 a	2.050 a
Available phosphorus/(mg·kg^−1^)	0.06370 a	0.06420 a	0.0650 a
Available potassium/(mg·kg^−1^)	11.35 a	11.53 a	11.61 a
Organic matter/(%)	15.27 a	15.28a	16.73 a
pH	7.680 a	7.700 a	7.831 a
Conductivity/(us·m^−1^)	11.88 a	11.79 a	11.76 a

Different letters indicate significant differences among slope positions (*p* < 0.05; least significant difference test).

**Table 3:** The density, plant height, and seed yield of giant ragweed (*Ambrosia trifida* L.) in the seedling period, growing period and mature period in 280 mm, 560 mm and 840 mm of simulated annual precipitation.

	Water Gradient	Seedling Period	Growing Period	Mature Period
Density/(individual/m^2^)	280 mm	68.3 ± 0.68 b	11.7 ± 0.66 c	3.89 ± 0.38 c
	560 mm	71.7 ± 0.76 b	30.5 ± 0.53 b	17.2 ± 0.54 b
	840 mm	74.4 ± 0.47 a	44.8 ± 0.84 a	37.8 ± 0.77 a
Plant height/cm	280 mm	16.2 ± 0.37 c	45.6 ± 0.66 c	63.9 ± 1.02 c
	560 mm	21 ± 0.56 b	85 ± 1.19 b	182 ± 3.76 b
	840 mm	27.2 ± 0.89 a	144 ± 3.33 a	259 ± 5.17 a
Seed yield/(individual/m^2^)	280 mm			19.1 ± 1.46 c
	560 mm			4661 ± 139 b
	840 mm			50,185 ± 2652 a

Different letters indicate significant differences for different water gradients (*p* < 0.05; least significant difference test).

## Data Availability

The original contributions presented in the study are included in the article; further inquiries can be directed to the corresponding authors.

## Supplementary Materials

The following supporting information can be downloaded at: https://www.mdpi.com/article/10.3390/biology14030249/s1, Table S1: Correlation data between soil physicochemical factors and biomass. Part of the research results of this study has been uploaded to Figshare. https://doi.org/10.6084/m9.figshare.28512764.

## Abbreviations

The following abbreviations are used in this manuscript:

GR	Giant ragweed
TS	Top slope
MS	Middle slope
BS	Bottom slope
